# Molecular mechanism of DRP1 assembly studied in vitro by cryo-electron microscopy

**DOI:** 10.1371/journal.pone.0179397

**Published:** 2017-06-20

**Authors:** Kaustuv Basu, Driss Lajoie, Tristan Aumentado-Armstrong, Jin Chen, Roman I. Koning, Blaise Bossy, Mihnea Bostina, Attila Sik, Ella Bossy-Wetzel, Isabelle Rouiller

**Affiliations:** 1 Department of Anatomy and Cell Biology, McGill University, Montreal, Quebec, Canada; 2 Facility for Electron Microscopy Research, McGill University, Montreal, Quebec, Canada; 3 Department of Biomedical Sciences, University of Central Florida, Orlando, Florida, United States of America; 4 Department of Molecular Cell Biology, Section Electron Microscopy, Leiden University Medical Center, Leiden, The Netherlands; 5 Department of Microbiology & Immunology, University of Otago, Dunedin, New Zealand; 6 Neuronal Networks Group, School of Clinical and Experimental Medicine, College of Medical and Dental Sciences, University of Birmingham, Birmingham, United Kingdom; Texas Technical University Health Sciences Center, UNITED STATES

## Abstract

Mitochondria are dynamic organelles that continually adapt their morphology by fusion and fission events. An imbalance between fusion and fission has been linked to major neurodegenerative diseases, including Huntington’s, Alzheimer’s, and Parkinson’s diseases. A member of the Dynamin superfamily, dynamin-related protein 1 (DRP1), a dynamin-related GTPase, is required for mitochondrial membrane fission. Self-assembly of DRP1 into oligomers in a GTP-dependent manner likely drives the division process. We show here that DRP1 self-assembles in two ways: i) in the presence of the non-hydrolysable GTP analog GMP-PNP into spiral-like structures of ~36 nm diameter; and ii) in the presence of GTP into rings composed of 13−18 monomers. The most abundant rings were composed of 16 monomers and had an outer and inner ring diameter of ~30 nm and ~20 nm, respectively. Three-dimensional analysis was performed with rings containing 16 monomers. The single-particle cryo-electron microscopy map of the 16 monomer DRP1 rings suggests a side-by-side assembly of the monomer with the membrane in a parallel fashion. The inner ring diameter of 20 nm is insufficient to allow four membranes to exist as separate entities. Furthermore, we observed that mitochondria were tubulated upon incubation with DRP1 protein in vitro. The tubes had a diameter of ~ 30nm and were decorated with protein densities. These findings suggest DRP1 tubulates mitochondria, and that additional steps may be required for final mitochondrial fission.

## Introduction

Mitochondria are dynamic organelles that frequently divide and fuse [1]. Mitochondrial division happens during cell division to distribute mitochondria to daughter cells and during apoptosis [2]. An imbalance between fusion and fission events leads to altered mitochondrial morphology that is implicated in aging and major neurodegenerative diseases including Huntington’s, Alzheimer’s, and Parkinson’s diseases [1, 3–5].

Members of the large GTPase dynamin superfamily, mechano-enzymes are responsible for mitochondrial fusion and fission [6]. Despite their functional diversity, all dynamin-related proteins (DRPs) are likely to undergo GTP-cycle-dependent conformational changes to drive self-assembly and disassembly [7–12]. Self-assembly is a required and rate-limiting step in membrane fission [13]. Mitochondrial fission requires DRP1 in humans and its homolog dynamin 1 (Yeast Dnm1) in yeast [14–16]. Mutations resulting from deletion or loss of function of Yeast Dnm1/DRP-1 lead to extensive mitochondrial networks and are indicative of an increase in mitochondrial fusion [14–19].

Dynamin 1 is the founding, and best characterized member of the dynamin superfamily at both the biochemical and structural levels [20–22]. Similar to Yeast Dnm1, the sequence-derived domain boundaries of DRP1 exhibit an amino-terminal GTPase domain that is followed by a middle domain (MID), and a GTPase effector domain (GED). It is, however, lacking the C-terminal proline and arginine-rich domain (PRD) and the membrane-binding pleckstrin homology domain (PH). For Yeast Dnm1, the MID and GED assemble into a bundle of four alpha helices called the stalk [22]. To form the bundle signalling element (BSE), three alpha helices, one from the GTPase domain, one from the middle domain and one from the GED domain come together [22]. The crystal structure of part of the *Arabidopsis thaliana* DRP1 indicates the N and C termini of the GTPase domain form a BSE domain [23]. In DRP1, the PH domain is replaced by a flexible region (or variable or B-insert; S1 Fig). While high-resolution structures of the GTPase domain have been solved, the structure of the B-insert domain (95 amino acids in the human isoform) remains unknown but is predicted to be unstructured [20, 24–28]. It has also been suggested the B-insert domain mediates binding to the membrane even though no densities were seen in the electron potential map of the Yeast Dnm1 [9]. While the B-insert domain may influence DRP1 self-assembly rate and/or geometry, it is not required for recruitment in the mitochondrial membrane, association with mitochondria, or for mitochondrial fission [29].

A tetramer under native conditions, dynamin 1 self-assembles into helical structures reminiscent of collars surrounding the neck of clathrin-coated vesicles [30, 31]. Dynamin forms helical tubes with an outer diameter of 50 nm that rely on PH domain interactions with negatively charged liposomes [7, 32, 33]. Addition of GTP to dynamin-lipid tubes leads to a 12 nm constriction of the lipid tubes, followed by the dissociation of the dynamin from the lipid bilayer [32]. Dynamin 1 and other dynamin family members have been shown to tubulate liposomes in a similar fashion [9]. Interactions with liposomes, as well as salt and nucleotide conditions, control the assembly and size of the oligomers formed by dynamin family members [30, 34–38]. Yeast Dnm1 forms spirals in the presence of non-hydrolyzable GTP analogs that are 110 nm in diameter in solution and 129 ± 40 nm when bound to lipids in the absence of nucleotide; they then constrict to 59 nm upon the addition of GTP [39]. Despite high sequence similarity, human DRP1 only assembles into ring-like oligomers in the presence of GTP and GMP-PNP, or spiral-like oligomers in the absence of lipids [4, 40]. The structural basis of DRP1 self-assembly and GTP-cycle-dependent conformational changes are incompletely understood. A possible mechanism of self-assembly has been derived from the crystal structure of the ‘stalk’ of interferon-inducible dynamin-like myxovirus-resistance protein 1 (MxA; also called MX1) in which the MID and part of the GED form an extended helical bundle that mediates self-assembly via conserved interfaces [41]. These interactions are conserved for dynamin 1 as shown by crystal packing [22]. Cryo-electron microscopy (cryo-EM) of structures of assembled dynamin in the presence of the non-hydrolysable GTP analogue GMPPCP, and in the absence of nucleotide, have provided models for the assembled oligomers, as well as the location of the GTPase and PH domains within the helical structures [22, 42].

Rosenbloom (2014) determined the diameter and length of the DRP1 helical rings encircling mitochondria during fission [43]. The mean lengths of the helical rings remained consistent during fission, but the significant decrease in diameters supported the twistase model of DRP1 constriction with potential loss of subunits at the helical ends [43]. During mitochondrial fission in the yeast *Saccharomyces cerevisiae*, Yeast Dnm1 is recruited to the outer mitochondrial membrane by the integral membrane protein Fis1 and the adaptor proteins Mdv1 and Caf4 [44–46]. The human ortholog of Fis1, hFis, is also required for complete fission of mitochondria [47]. The mammalian adaptor proteins homologous to Mdv1 and Caf4 have yet to be identified. An investigation of the role of MiD49 and MiD51 in mitochondrial fission found the two receptors on the mitochondrial outer membrane recruit DRP1 to facilitate mitochondrial fission [48].

Studies of the mitochondria-specific phospholipid, cardiolipin (CL), have shown it promotes oligomerization and stimulation of DRP1 GTPase activity as well as membrane tubulation [49–51]. Ugarte-Ugribe (2014) studied the ability of full-length DRP1 to constrict lipid bilayers through a GTP hydrolysis-dependent mechanism [52]. The mitochondrial fission factor (Mff), a tail-anchored membrane protein of the mitochondrial outer membrane, recruits DRP1 to sites of fission. The differential stimulatory effects of Mff on disparate DRP1 isoforms were found to be dependent on cardiolipin [53]. Mff also regulated the auto-inhibitory effect imposed by these sequences on DRP1 function.

One of the four distinct domains in the DRP1 protein, the variable domain, was found to limit premature DRP1 assembly in solution and promote membrane curvature. The mechanochemical core of DRP1, without the variable domain, was sufficient to mediate GTP hydrolysis-dependent membrane constriction [53]. A recent study demonstrated that dimers and not multimers heighten the reassembly and reorganization of DRP1 for mitochondrial membrane remodeling both in vitro and in vivo [54]. DRP1 self-assembly in solution impairs functional interactions with membrane-anchored Mff. Instead, dimeric DRP1 species were selectively recruited by Mff to initiate assembly of a functional membrane fission complex. Taken together, these findings provide a mechanism wherein the multimeric states of both Mff and DRP1 regulate their collaborative interaction.

Although the structure of human DRP1 dimer was resolved by X-ray crystallography and cryo-EM [55], the dynamics and higher-order assembly of DRP1 are incompletely characterized. To understand the basis of human DRP1 self-assembly and its mechanism of action, we determined a three-dimensional (3D) electron microscopy map of the ring-like oligomers assembled by DRP1 in the presence of GTP and absence of lipids using cryo-EM and single-particle analysis (SPA). The helical oligomers obtained in the presence of GMP-PNP, a non-hydrolysable GTP analog were characterized using Electron Tomography (ET). We docked the human dynamin GTPase domain and the MID/GED domains of MxA into the DRP1-GTP map. Combining this information, we propose a new mechanism of DRP1 oligomerization.

## Materials and methods

### Cloning, expression and purification of human DRP1 protein in bacteria

Human muscle-specific DRP1 isoform 3 was expressed and purified as previously described [4, 40]. Briefly, the pET21 plasmid containing the human DRP1 sequence (NCBI accession number: NM_005690.3, with 699 amino acid residues plus C-terminus H-6 His tag) was expressed in *E*. *coli* BL21 (DE3) star cells (Stratagene). Protein expression was induced at 25°C overnight with 0.5 mM IPTG. Cells were harvested by centrifugation and resuspended in 50 mM KH2PO4, 300 mM NaCl, 5 mM imidazole, 0.1 mg/ml lysozyme, and 10% glycerol (pH 7.8). Cell lysis was performed by sonication. Soluble 6×-His-tagged proteins were loaded onto Ni-NTA resin, washed with 20 mM Tris, 300 mM NaCl, 40 mM imidazole, 10% sucrose, and 10% glycerol (pH 7.8). The bound protein was eluted with 25 mM Tris, 300 mM NaCl, 500 mM imidazole, and 10% glycerol (pH 7.8). The DRP1 containing fractions were injected onto a Superdex 200 10/300 GL ÄKTA FPLC column (GE Healthcare) equilibrated with 30 mM Tris, 100 mM NaCl, 2 mM DTT, and 1 mM EDTA (pH 7.8). Purified proteins were dialyzed in 30 mM Tris and 100 mM NaCl (pH 7.8) and then stored at –80°C.

### Negative stain transmission EM

Purified DRP1 was kept overnight in the presence of 1mM DTT at 4°C. Incubation was typically performed for 4 hours on ice at a DRP1 concentration of 2.3 mg/mL with 2 mM MgCl_2_ in the absence or presence of 2 mM nucleotide GTP, GDP, or GMP-PNP (Sigma-Aldrich). Samples incubated with GTP and GMP-PNP were further diluted by a factor of ~50 x in 25 mM HEPES, 25 mm PIPES, 2 mM MgCl_2_ and 2 mM GTP, (pH 7.8) and 25 mM HEPES, 25 mm PIPES, 2 mM MgCl_2_ and 2 mM GMP-PNP, and 150 mM NaCl (pH 7.8), respectively. The samples were pipetted onto glow-discharged, carbon-coated TEM grids and stained with 2% uranyl acetate (SPI Supplies) and imaged with an FEI Tecnai 12 TEM (FEI) at an accelerating voltage of 120 kV and equipped with an AMT XR80C CCD camera (Advanced Microscopy Techniques, NJ).

### Cryo-EM

Five μL of the assembled protein was pipetted onto glow-discharged Quantifoil holey carbon TEM grids (Electron Microscopy Sciences). Excess fluid was removed before the sample was flash frozen hydrated by plunging into a bath of liquid ethane slush using the FEI Vitrobot Mark IV [56]. The grids were stored in liquid nitrogen until imaged with the Tecnai G^2^ F20, operated at an accelerating voltage of 200 kV or Titan Krios cryo-STEM (FEI) at an accelerating voltage of 300 kV. Images were recorded under low dose conditions with a Gatan Ultrascan 4000 CCD camera at a nominal magnification of 50,000 X corresponding to a pixel size of 0.22 nm and a defocus level ranging from − 1.5 to − 2.5 μm.

### Single particle 3D reconstruction

Using Signature software, ring-like GTP-bound DRP1 particles with minimum astigmatism and drift were selected from the digital images [57]. The contrast transfer function (CTF) was estimated using CTFFIND3 [58]. 2606 particles were manually selected from the images and out of these, 786 well defined rings were analyzed using k-means classification of the SPARX software suite [59]. K-means was performed with a small number of classes followed by several rounds of multi-reference alignment to assess the composition homogeneity of the oligomers. To calculate the 3D EM map, we only included rings that contain 16 monomers (147 rings or 2352 asymmetric subunits). The initial model was obtained using the common line method from the most characteristic averages obtained for the 2-D classification. The EM map was refined in SPARX with C16 symmetry imposed. Chimera was used for the final 3D visualization, interpretation and manual docking of the high-resolution structures solved by X-ray crystallography [27, 60]. Since, the GTPase domain of DRP1 has higher similarity to the GTPase domain of Human dynamin 1 than to the *Arabidopsis thaliana* DRP1 GTPase domain (S1 Table), we used the human dynamin 1 GTPase domain to assign the electron potential corresponding to the GTPase domain (PDB ID: 3SNH) in our EM map. As the DRP1 MID and GED domains have higher sequence similarity to the MxA stalk (S1 Table), we used the MxA stalk (PDB ID: 3LJB) to locate the DRP1 MID/GED (PDB ID: 3LJB) domains in our EM map.

### Labelling of DRP1 C-terminus with Ni-NTA-Nanogold

The DRP1 oligomeric rings were formed after overnight incubation with 1mM DTT at 4°C, followed by 4 hours of incubation on ice at a DRP1 concentration of 2.3 mg/mL with 2 mM MgCl_2_ and 2 mM GTP. The protein was diluted to 50 μg/mL in 25 mM HEPES-PIPES buffer containing 2 mM MgCl_2_ and 2 mM GTP. The DRP1 oligomers were incubated for one minute on a TEM grid (for negative stain TEM) or Quantifoil holey carbon TEM grid (for cryo-EM) and fixed with 4% paraformaldehyde (PFA) for one minute. Five uL of 2.8 nm Ni-NTA-Nanogold (Nanoprobes) was pipetted onto the grids and incubated for 30 minutes in a humidified chamber at room temperature. The grids were washed five times with 25 mM HEPES buffer and immediately plunge-frozen in liquid ethane using the Vitrobot. For negative staining, the grids incubated with Ni-NTA-Nanogold were washed 5 times with 25 mM HEPES, 25 mM PIPES (pH 7.8) buffer and stained with NanoVan (Nanoprobes). Images of the NanoVan-stained grids were collected with both the FEI Tecnai 12 TEM at 120kV and FEI Titan Krios cryo-STEM at 300 kV. Additional experiments with and without 40 nM Immidazole were carried out on the Drp1 rings and helices labelled with Nanogold. This was done to remove non-specific binding of Nanogold (S2 Table). The selection of particles and the 3D single particle reconstruction were undertaken as described above, including 458 Nanogold labelled cryo images of rings. No symmetry was applied.

### 3D characterisation of DRP1 helices using electron tomography

Tomograms of the GMP-PNP-bound DRP1 helices attached with Ni-NTA-Nanogold were acquired with the FEI Tecnai G^2^ F20 cryo-STEM at 200 kV. Images were recorded at a nominal magnification of 25,000X corresponding to a pixel size of 0.48 nm (for the NanoVan-stained samples). The tomograms for DRP1 helices unbound to the Nanogold were collected with the FEI Titan Krios cryo-STEM at 300 kV using a tilt range between −60° to 60° at 18,300 fold magnification corresponding to a pixel size of 0.820 nm. The images were aligned, cropped, and binned using IMOD [61–63]. One representative tomogram (acquired in cryo condition), binned twice to 1.64 nm/pixel, was manually traced in 3dmod, Version 4.5 [60]. Chimera was used to compare the DRP1 model helices to the extracted helix data traced in 3dmod. Additionally, the pitches and diameters of the helices were manually measured on the digital images.

### 3D characterisation of DRP1 rings using electron tomography

Tomograms of the GTP-bound DRP1 rings labelled with Ni-NTA-Nanogold were collected with the FEI Tecnai G^2^ F20 cryo-STEM at a nominal magnification of 25,000X corresponding to a pixel size of 0.44 nm (with negative-stained samples) using tilt range between −60° to 60°. The images were aligned, cropped, and binned using the IMOD [61]. The 3D visualization and interpretation was carried out with Chimera [27, 60].

### Mitochondria purification

All animal studies were carried out at the Montreal Neurological Institute following the guidelines of the Canadian Council on Animal Care. Two wild type C57BL/6 mice were sacrificed by cervical dislocation and their livers were rapidly removed for mitochondrial extraction [64]. This method allows the preparation of two pure mitochondrial populations (heavy and light mitochondria) with high yield [65]. In this study, we used the light population of mitochondria (0.5-μm diameter). 1 mg/mL of mitochondria were incubated with DRP1 at 50 μg/mL in the presence of 2 mM GMP-PNP in 25 mM HEPES, 25 mM PIPES, 2 mM MgCl_2_, and 150 mM NaCl (pH 7.8). Mitochondria without DRP1were negative controls.

## Results

### GTP binding promotes DRP1 self-assembly

**Fig 1:**
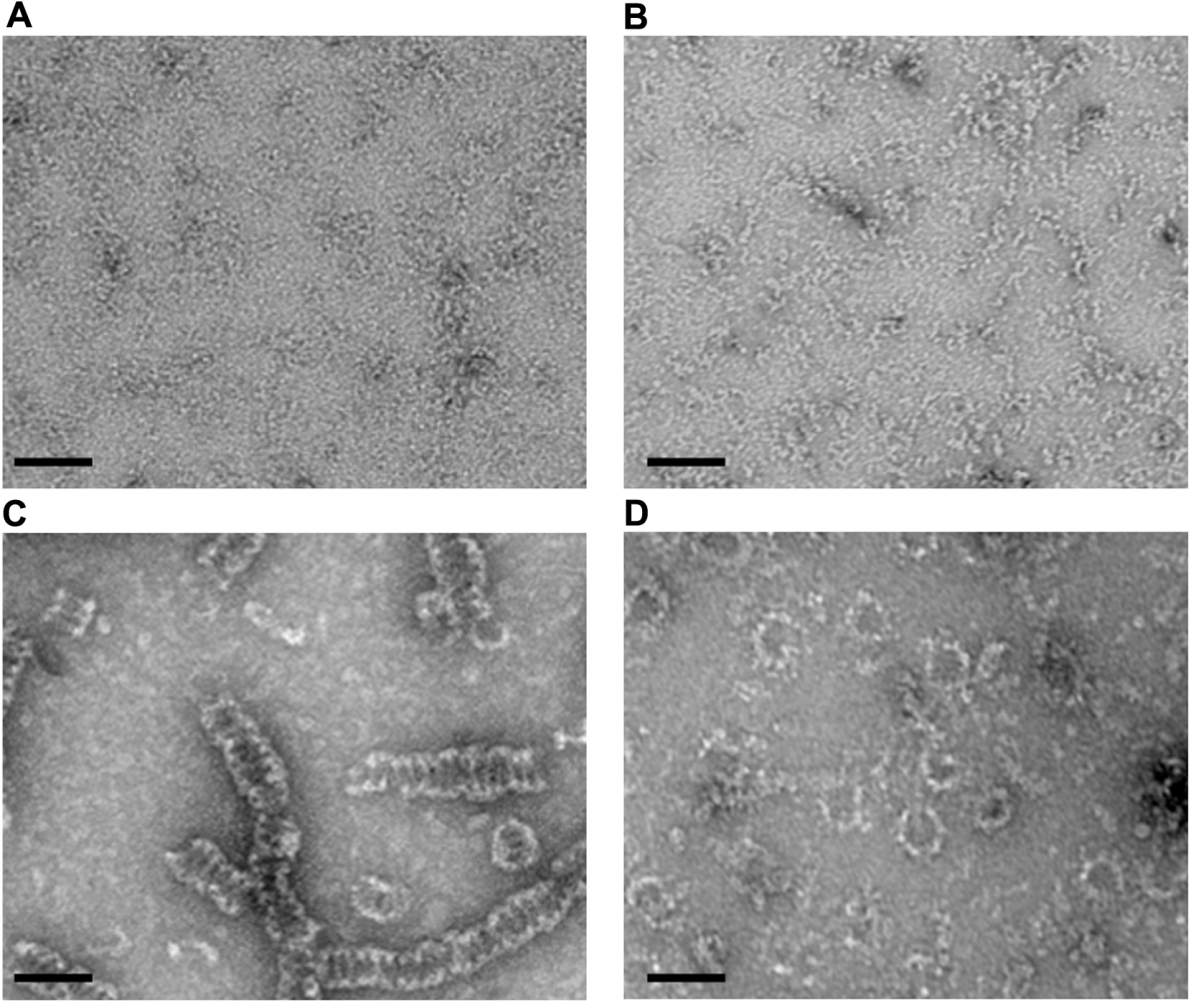
DRP1 ring- and spiral-like structures by negative-stain TEM. Negative-stained TEM images of DRP1 in the absence of nucleotide (A) or in the presence of GDP (B), GMP-PNP (C), or GTP (D). Scale bar = 50 nm).

Negative stain TEM of the purified muscle-specific isoform 3 of human DRP1 confirmed the assembly into ring-like and spiral-like oligomers, as previously described [4, 40]. In the absence of nucleotide or in the presence of GDP, DRP1 did not self-assemble under any salt condition tested (5mM to 150mM NaCl (Fig 1A and 1B). In the presence of GMP-PNP, DRP1 self-assembled into spiral-like structures with a diameter slightly larger than that of the rings (~36 nm) of variable length, reaching up to several microns (Fig 1C). In the presence of GTP, however, DRP1 self-assembled into ring-like oligomers composed of 13 to 18 monomers. Each ring has a flexible diameter depending upon the number of monomers. The most abundant rings were composed of 16 monomers having outer and inner ring diameters of ~30 nm and ~20 nm, respectively (Fig 1D). The 3D analysis was performed with rings containing 16 monomers because they were slightly more abundant (Fig 1D).

### 3D electron microscopy map of the human DRP1 rings

**Fig 2:**
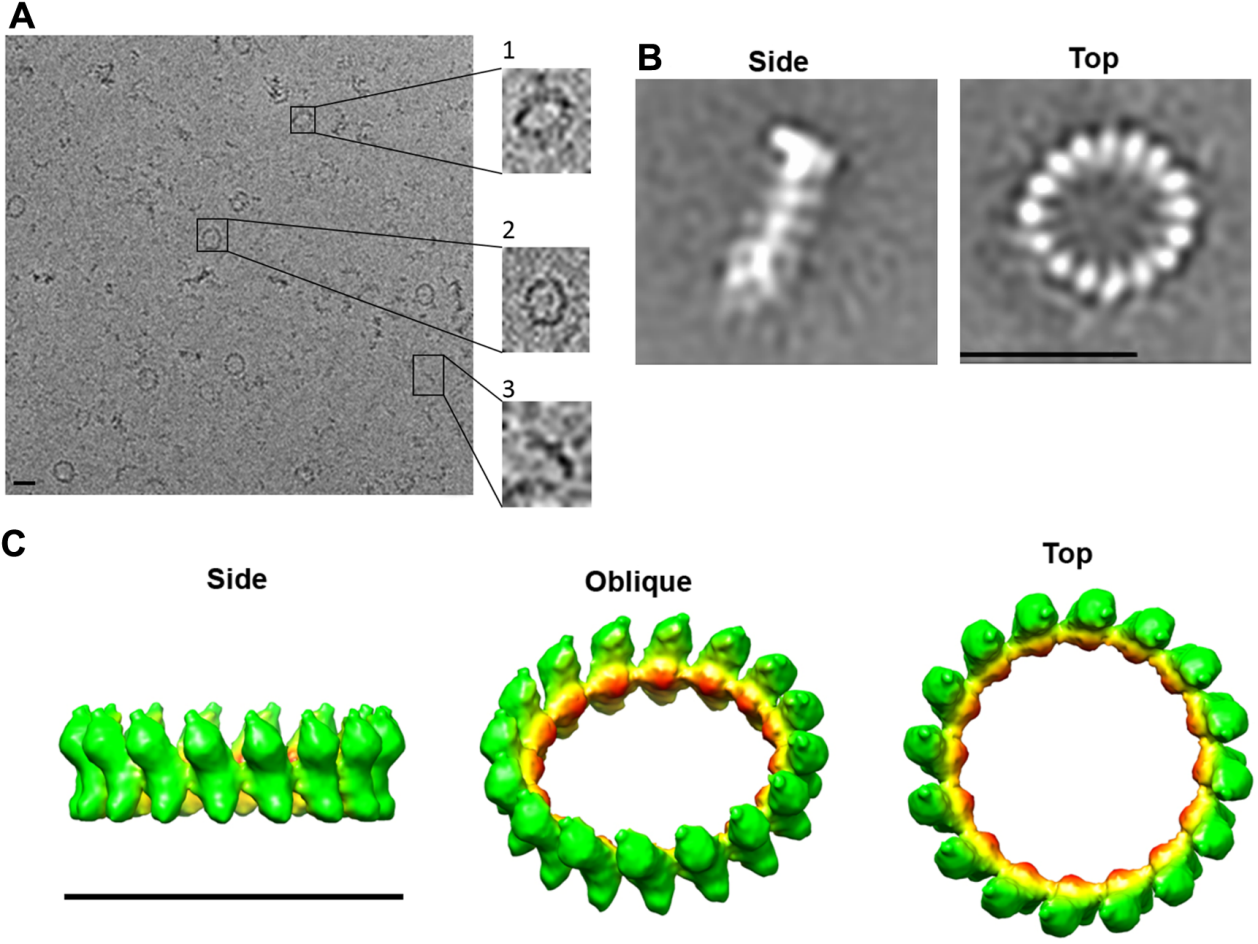
3D structure of human GTP-bound DRP1 ring-like oligomers. (A) A representative cryo-EM CCD image of GTP-bound human DRP1. The inserts show examples of top, side and intermediate views (Scale bar = 30 nm). (B) The two most characteristic class averages showing the DRP1 -GTP rings on its side and as seen from the top. The contrast has been inverted for clarity purposes in respect to (A) (Scale bar = 30 nm). (C) Isosurface 3D representation of the electron density map of GTP-bound DRP1 ring-like structures by single particle image analysis. The map displayed by Chimera has been radially coloured. The estimated resolution according to the Fourier shell correlation of 0.5 is ~25Å (Scale bar = 30 nm).

We calculated the electron microscopy map of the GTP-bound DRP1 rings using cryo-EM and single particle reconstruction (Fig 2A). The two most characteristic classes obtained after K-mean image analysis are shown in (Fig 2B). K-mean analysis showed the oligomers have a unique size comprising of 16 monomers with some exceptions of 13–18 monomers (S2 Fig). The average made from the group containing 16 monomers showed rings in the top view orientation have an outer diameter of ~30 nm and an inner diameter of ~20 nm. The average of rings in the side view orientation shows a height of ~10 nm (Fig 2B). The 3D reconstruction is in agreement with the dimensions measured in the 2-D analysis and clearly confirms the oligomeric rings were mostly composed of 16 DRP1 monomers with a distance of 5 nm between the center of gravity of two adjacent monomers (Fig 2C). The monomers are connected by a central region. The resolution of the resulting oligomeric model complex was ~25 Å according to the Fourier shell correlation criteria of 0.5.

### Domains assignment to the 3D map of the GTP-bound DRP1 rings

**Fig 3:**
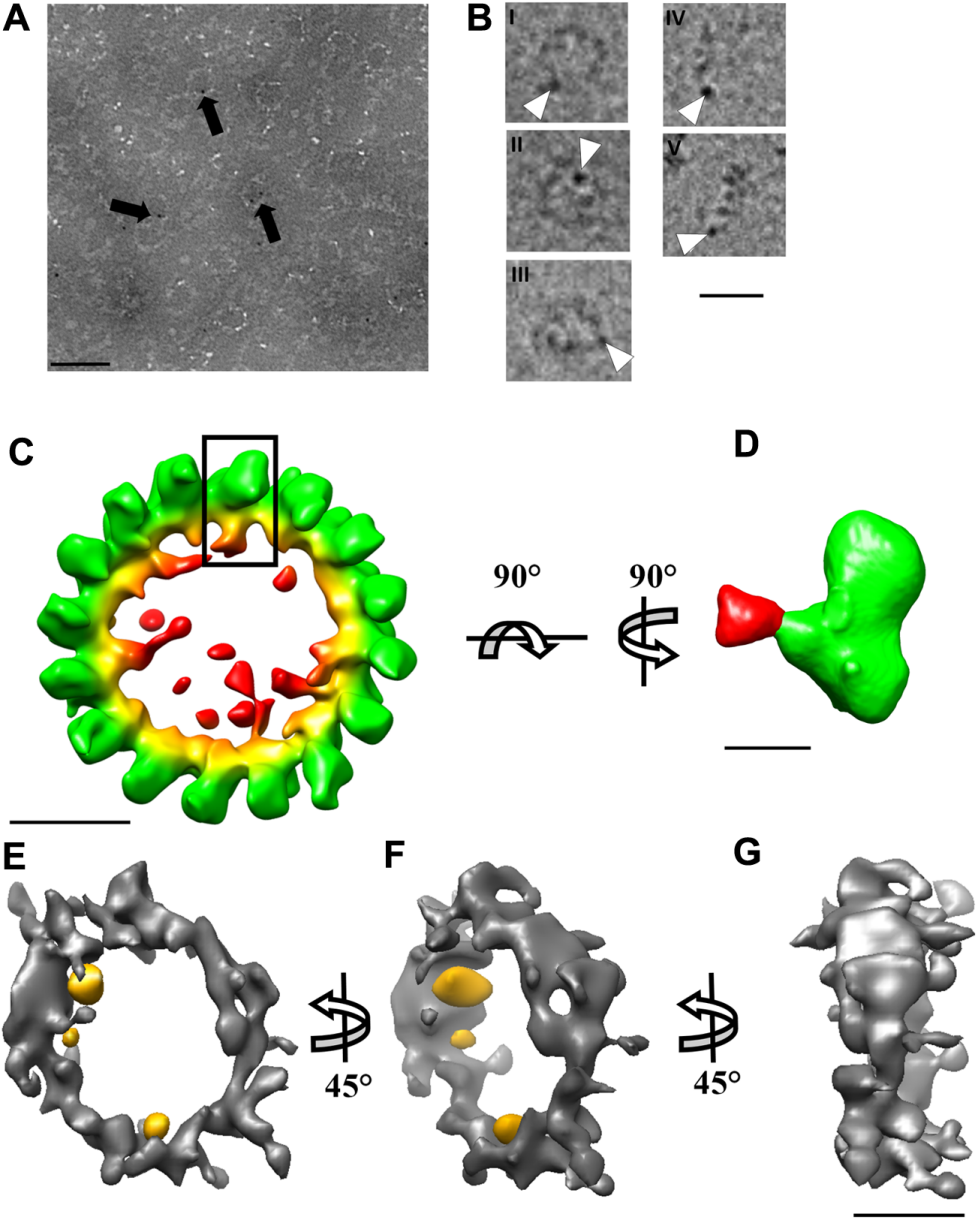
Localization of the C-terminus of DRP1 using Ni-NTA Gold labeling. (A) TEM image of DRP1-GTP rings labelled with Nanogold-NTA (black arrows) and negatively stained with NanoVan (Scale bar = 50 nm). (B) Cryo-TEM image of DRP1-GTP rings labelled with Nanogold (white arrowheads). The rings are shown in the top (i & ii), oblique (iii) and side views (iv & v) (Scale bar = 30 nm). (C) Radially-coloured isosurface representation of the 3D electron density map of the DRP1-GTP rings labelled with Nanogold (Scale bar = 10 nm). (D) Reconstructed monomer extracted from the boxed area of the Nanogold-bound DRP1-GTP ring (C) was rotated 90° about the axis to show the monomer map (Scale bar = 5 nm). (E), (F), (G) Isosurface representation of the Nanogold-bound DRP1-GTP rings reconstructed from the tomogram. (E) and (F) show 45° rotation about the y- axis (Scale bar = 10 nm).

Ni-NTA-Nanogold was used to label the C-terminal His_6_-tag of the DRP1 oligomers and the ring complex viewed using negative stain TEM (Fig 3A and 3B). Without fixing the DRP1 rings in paraformaldehyde, the addition of Ni-NTA-Nanogold destabilized the rings. One or two gold particles were observed per DRP1 ring. This suggests that not all the C-terminal His_6_-tags were labelled (Fig 3C). To avoid flattening artefacts while locating the Nanogold within the 3D map, we processed the labelled rings for cryo-EM (Fig 3D). Ni-NTA-Nanogold-labelled oligomeric particles comprised of top, side, and intermediate views were selected. A K-means analysis of the ring-like structures verified the labelled oligomers were homogeneous rings composed of 13 to 18 monomers of variable diameter. The most abundant oligomers were composed of 16 monomers with a diameter of 30 nm. A 3D reconstruction of the labelled rings was calculated without imposing symmetry The position of the Nanogold in the 3D volume of a single monomer of DRP1 was found to be in the middle between the top globular domain assigned to the GTPase domain and the lower elongated lobe assigned to the middle/GED domains (Fig 3D). Isosurface representation of the Nanogold-labelled DRP1-GTP rings reconstructed from a tomogram and rotated 45° about the y-axis showed the labelling was limited to the inner diameter of the rings (Fig 3E, 3F and 3G).

**Fig 4:**
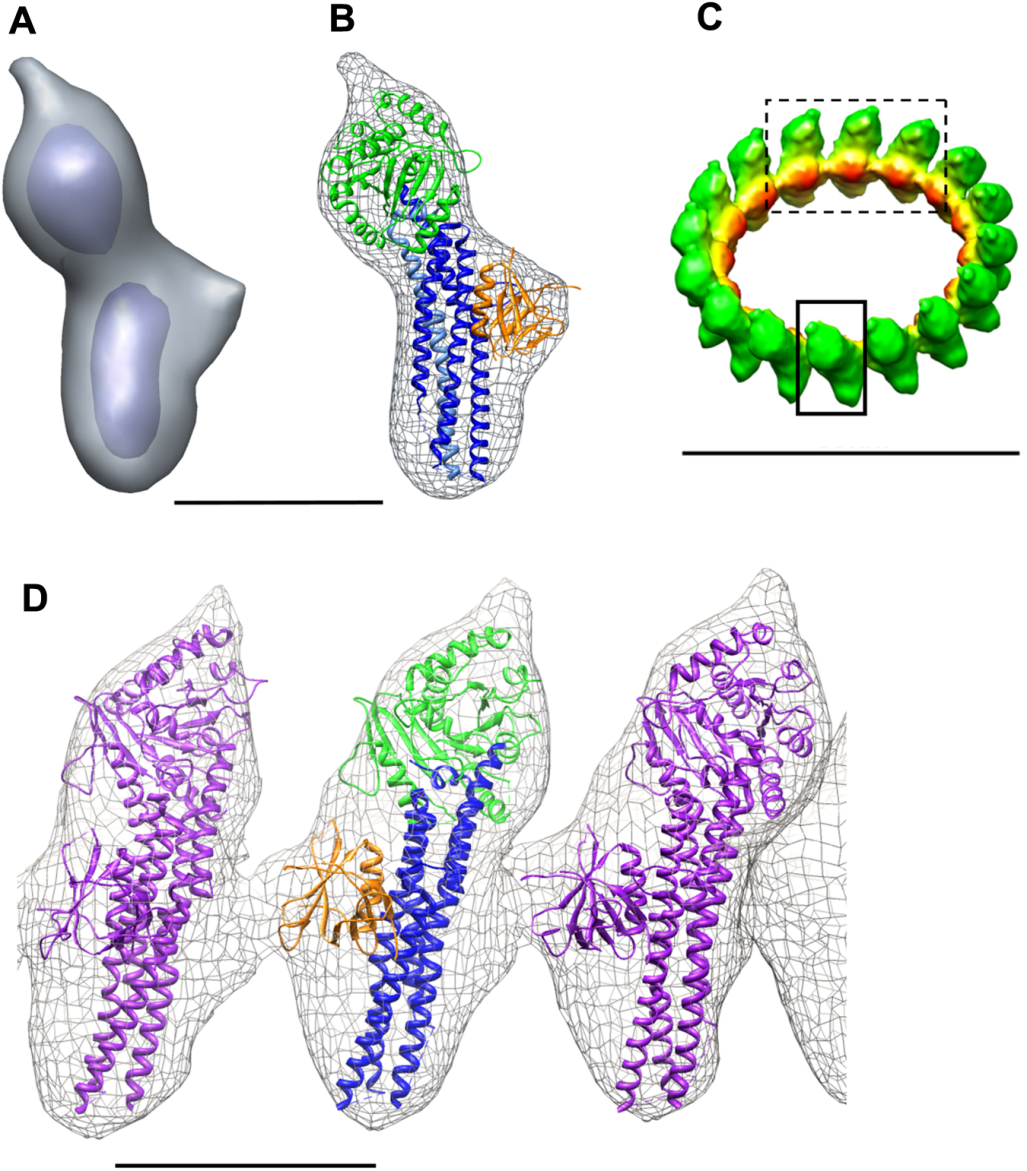
Domains assignment to the electron density map of the GTP-bound DRP1 ring-like oligomers. (A) Electron density map of a monomer segmented from the map (small box (C)). The monomer is shown as two contour levels to emphasize the globular shape of the top domain and the elongated shape of the bottom domain (Scale bar = 5 nm). (B) The high-resolution structures of human dynamin GTPase domain (pdb id: 3SNH, shown in green as a cartoon representation) and the human MxA stalk Middle-Effector Domain (residues 366−636, pdb id: 3LJB, shown in dark blue and light blue as a cartoon representation) were docked into the EM map of a segmented monomer (shown in grey chicken wire) using Chimera. The EM map contains additional densities that cannot be accounted for by the GTPase and the MID/GED domains. These are likely to correspond to the position of the BSE and B-insert domains. For size estimation only, we have introduced the PH domain of dynamin (residues 518−631, pdb id: 3SNH) (Scale bar = 5 nm). (C) EM map of GTP-bound DRP1 in an oblique view (Scale bar = 30 nm). (D) The domains in (B) are shown for the three adjacent monomers (dashed box (C)) (Scale bar = 5 nm).

Each monomer contained two distinct main densities: a globular and an elongated density (Fig 4A). On the basis of sequence similarity, the human dynamin1 GTPase domain (pdb id: 3SNH; 59% identity and 79% similarity with human DRP1) was docked into the globular domain of the monomer, while the MID/GED stalk structure of MxA (residues 366 to 636, pdb id: 3LJB) was docked into the elongated domain (S1 Table). We chose to dock the MxA stalk domain because the sequence similarity between the DRP1 MID/GED domains and the MxA domains is higher than between the DRP1 MID/GED domains and the human dynamin MID/GED domains (S1 Table). There was good agreement between the size of the domains solved at high resolution and the EM map (Fig 4B and 4C). Moreover, there was no additional space in the height of the monomers. The EM map contains additional densities that cannot be accounted for by the GTPase and MID/GED domains in the width of the monomer. These are likely to correspond to the position of the BSE and B-insert domain. This positioning suggests that the location of these domains will be at the border of each monomer, possibly allowing interaction between subunits. For estimation of the size of the additional density only, we docked the high-resolution structure of the Yeast Dnm1 PH domain into the EM density (Fig 4B and 4D).

The position of the Nanogold attached to the C-terminus of DRP1 is compatible with our docking, as it places the C-terminus of DRP1 in the central region (Fig 3E, 3F and 3G).

### Characterization of DRP1-GMP-PNP helices

**Fig 5:**
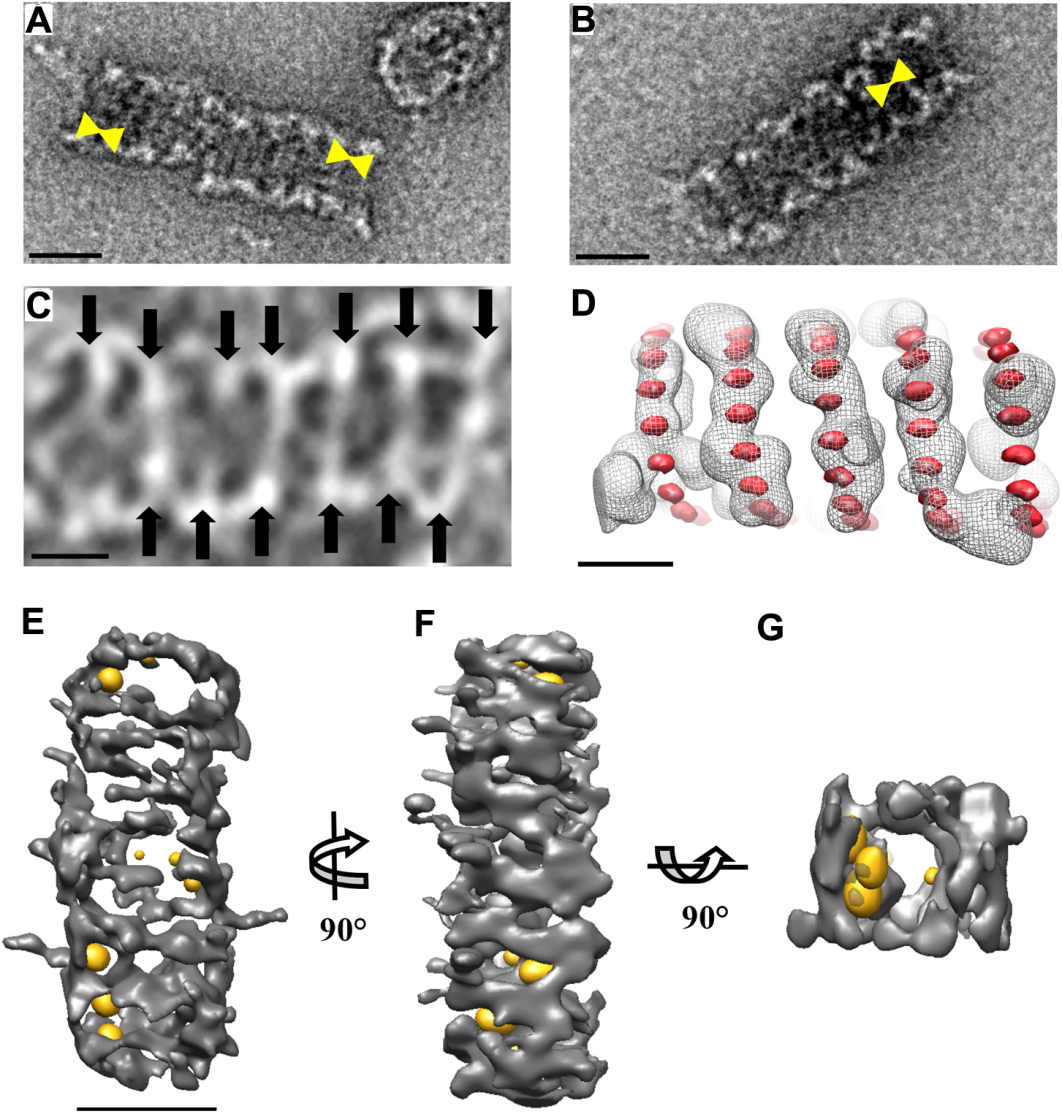
3D Analysis of GMP-PNP-bound DRP1 helices. (A−B) TEM images of DRP1-GMP-PNP helices after negative staining with NanoVan. The mean pitch of the helices is 13.7 nm (yellow markers; Scale bar = 30 nm). (C) Image represents the average of 20 slices from the tomogram of DRP1-GMP-PNP helix in cryo-ET. The contrast has been inverted for viewing purposes. Protein density is shown in white. Black arrows show the helical turns (Scale bar = 15 nm). (D) A filtered-traced helix traced from the cryo-tomogram of a DRP1-GMP-PNP helix (grey chicken wire). The position of the monomers with an ideal helix of 30 nm in diameter and a pitch of 13.7 nm (Red dots; Scale bar = 15 nm). (E), (F), (G) Isosurface representation of Nanogold-bound DRP1-GMP-PNP helices reconstructed from the tomogram with (E) rotated 90° about the y-axis; (F) rotated 90° about the x- axis, and (G) cross-sectional view showing the gold labelled inside the helix. The map displayed by chimera has been low pass filtered to ~30Å (Scale bar = 30 nm).

Electron tomography of both negative-stained and frozen-hydrated specimens showed that helices assembled in the presence of GMP-PNP were of variable length (Fig 5A and 5B). We measured the pitch and diameter of several helices as well as their variations within a single helix in the negative-stained images using 3dmod. Seventy-one pitch measurements were taken in total, occasionally with two from the same helix, resulting in a mean pitch of 13.7 nm, a median pitch of 13.73 nm, and a standard deviation of 1.7 nm. The pitch measurements were confirmed using tomograms of the negative-stained helices. We observed, however, a slight variation in the diameter of the helices, both between helices and within the same helix. The average outer diameters of the helices were 36.28 nm with a standard deviation of 3.51 nm.

Tomograms collected from cryo-ET were also calculated, as cryo-EM avoids possible flattening from drying artefacts, enabling better preservation of the helices in their native state. A few helices were also analyzed from the cryo-tomogram. The model helix was matched to the pitch and diameter shown in two cryo-tomograms (Fig 5C and 5D). Densities from the cryo-tomogram were low-pass filtered and manually traced using 3dmod. To search for parameters matching the data, the variations in diameter and pitch of the model helix were performed. The parameters shown in (red dots) correspond to a diameter of 30 nm and a pitch of 13.7 nm (Fig 5D). As in negative stain TEM, the diameter of the helices in cryo-ET tended to be of variable length, even along a single helix. The measurements done from the cryo samples correlated well with data collected in negative stain TEM, suggesting minimal deformation. The cryo-tomograms of Nanogold-labelled DRP1-GMP-PNP helices were also reconstructed (Fig 5E, 5F and 5G). The 3D map revealed that the Nanogold was labelled on the inner face of the helices, confirming the presence of the C-terminus within the inner face.

### Binding of DRP1-GMP-PNP to mitochondria

**Fig 6:**
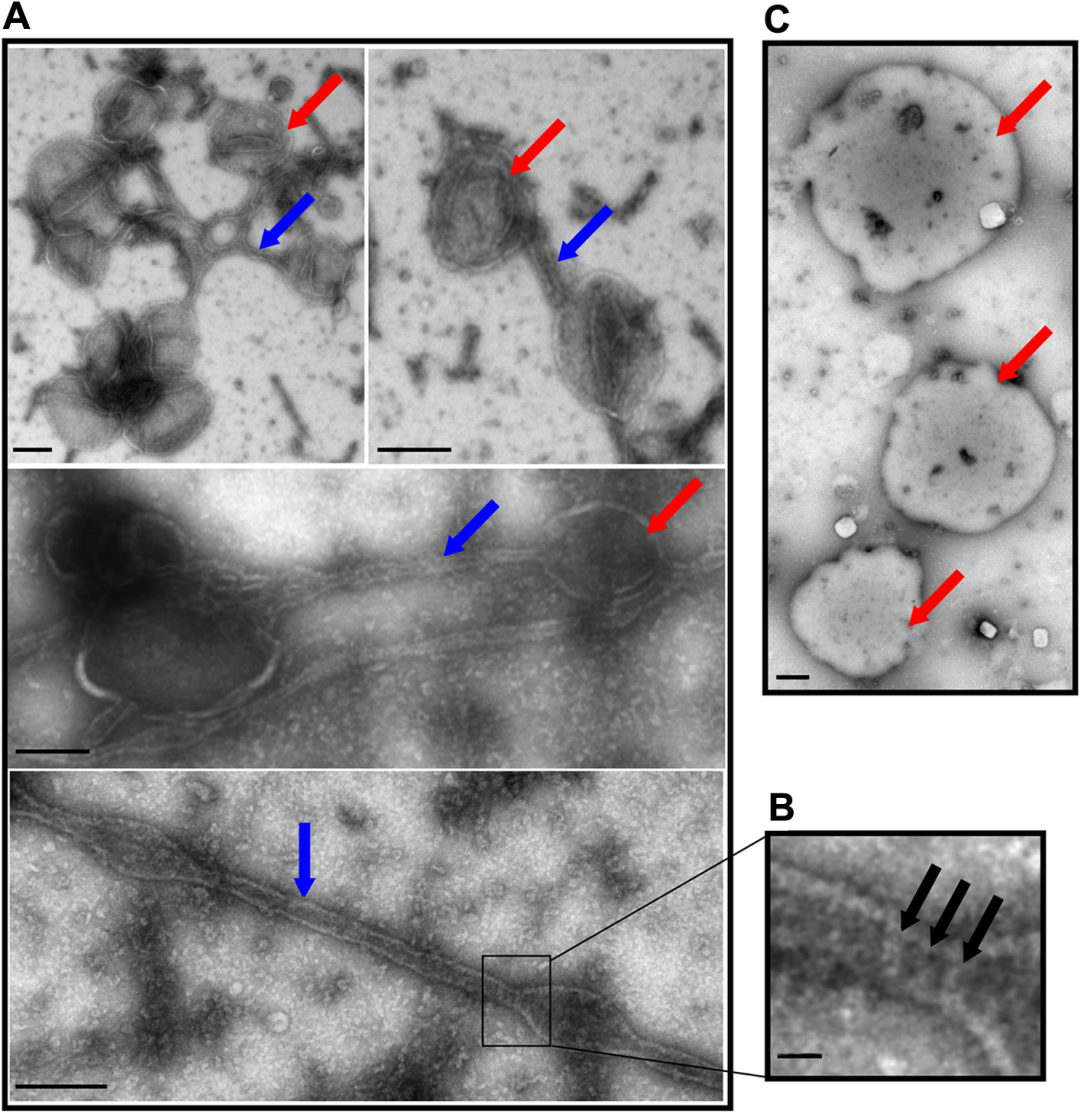
Binding DRP1 to mitochondria in the presence of GMP-PNP by negative-stain EM. (A) Negative-stain TEM image of mitochondria (red arrows) and mitochondria tubules (blue arrows) in the presence of DRP1-GMP-PNP (Scale bar = 100 nm). (B) TEM image shows three turns of the helices forming the mitochondria tubules (black arrows) (Scale bar = 30 nm). (C) Negative-stain TEM image of mitochondria control (without DRP1). (Scale bar = 100 nm).

DRP1 was added to mitochondria in presence of GMP-PNP. It shows the formation of mitochondria tubules corresponding to DRP1 oligomers around the mitochondria (Fig 6A). Three bands of protein density wrapping around a tubule, suggests that DRP1 may be making a set of rings or a short helix around the mitochondria to mediate fission (Fig 6B). Negative controls corresponding to mitochondria without DRP1 did not show any tubules (Fig 6C).

## Discussion

In the current study, we present the 3D structure of DRP1 oligomeric ring in the presence of GTP and the characterization of the helices assembled in the presence of GMP-PNP. The assembly process required magnesium; the formed structure is dependent on GTP or GMP-PNP. In particular, in the absence of nucleotide and in the presence of GDP, DRP1 fails to self-assemble. By contrast, GTP binding stimulates DRP1 self-assembly into a ring-like structure composed of 13 to 18 monomers. The most abundant rings were composed of 16 monomers having outer and inner ring diameters of ~30 nm and ~20 nm, respectively (Fig 2). The data suggest that human DRP1 spontaneously self-assembles into oligomeric structures dependent on GTP, consistent with previous reports [4, 13, 66]. In the presence of GMP-PNP, DRP1 self-assembles into spiral-like structures with variable length and a diameter similar to the rings (~36 nm), but with a constant pitch of ~13.7 nm. The variation in diameter suggests that helices have some flexibility to adapt to the size of the mitochondria. This flexibility is, however, lost in the presence of GTP as the rings may have formed with different diameters owing to the inclusion of additional monomers. It is possible that GTP molecules are being hydrolysed in the ring conformation, inducing loss of flexibility.

**Fig 7:**
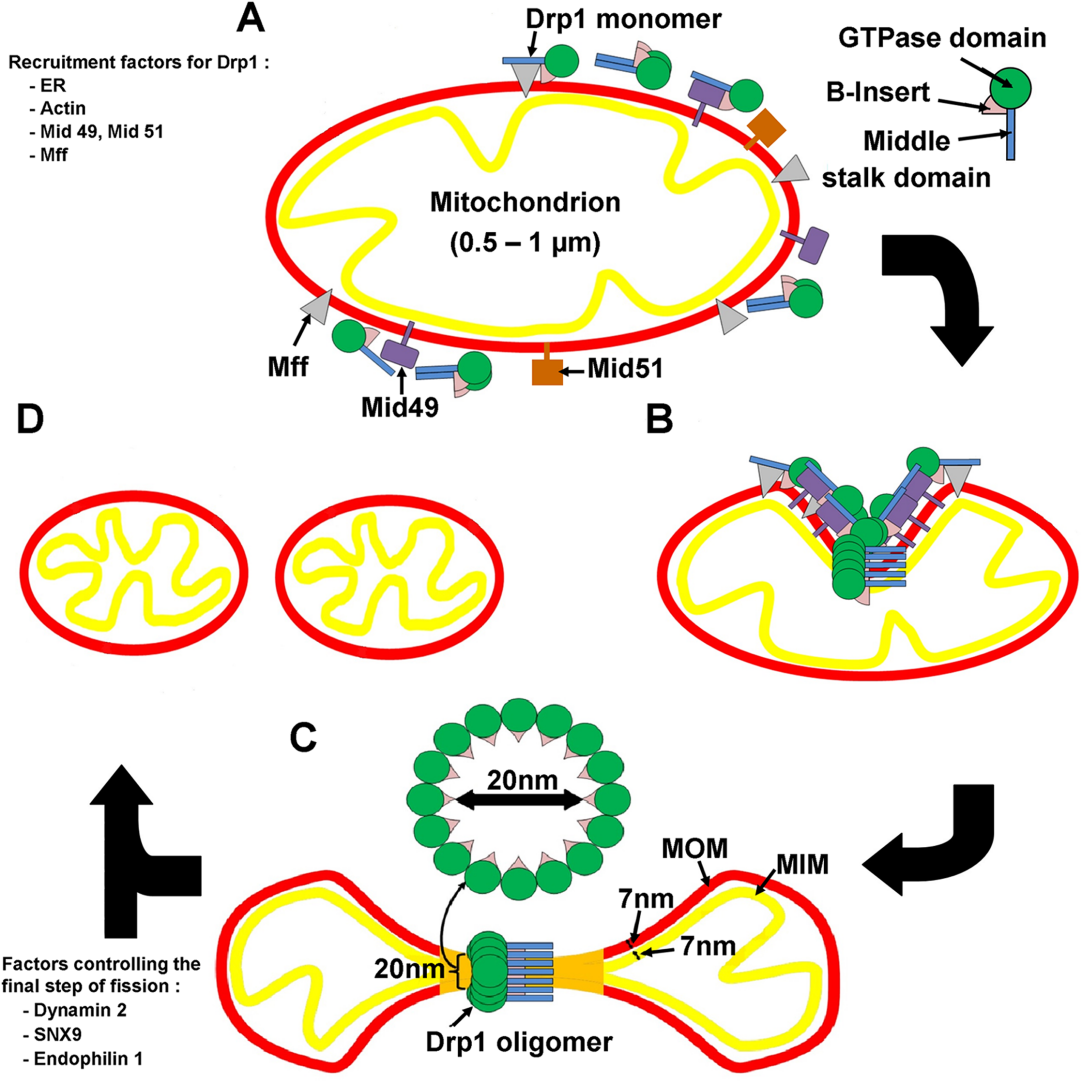
Model showing the mechanism of DRP1 in mitochondrial fission. DRP1 monomers or dimers are recruited from the cytosol by recruitment factors including Mff and Mid49. They assemble into spiral/rings shaped oligomers of 30 nm outer diameter and inner diameter of 20 nm. As the thickness of both inner and outer mitochondria membranes are 7 nm [67], assembly of DRP1 onto the mitochondria would simultaneously constrict the mitochondria in such a way that four independent membranes would not be able to exist as separate units inside the DRP1 rings (Stage C; orange area).

More importantly, the most abundant DRP1 GTP-bound rings were composed of 16 monomers and had an inner diameter of 20 nm, which is insufficient to surround four mitochondrial membranes. Since the thickness of the inner and outer mitochondrial membranes have each been reported to be 7 nm, four membranes (two inner and two outer) cannot exist as separate entities within the lumen of the DRP1 rings (Fig 7) [67]. The apparent rigidity of the DRP1 rings in the presence of GTP suggests GTP oligomerization is sufficient to trigger a membrane fusion event. The oligomers that were obtained were significantly smaller than those having dynamin 1 or Yeast Dnm1 bound to the lipids. Dynamin assembles on lipids into helices with an outer diameter between 40−50 nm and an inner lipid lumen between 7−17 nm [42, 68]. Yeast Dnm1 assembles on lipids into much larger oligomers with an outer diameter of 129 nm and an inner lipid lumen diameter of 89 nm. These constrict upon GTP hydrolysis to an outer diameter of 67 nm and inner lipid lumen of 25 nm [9].

The difference between human DRP1 and Yeast Dnm1 is particularly striking given their high amino acid similarity. In the absence of lipids, human DRP1 helices form a 1-start turn similar to dynamin 1, while Yeast Dnm1 assembles into 2-start helices in the presence of lipids [9]. In the helical reconstruction of Yeast Dnm1 onto lipids, the general shape of a monomer is similar in its globular and elongated domain to that which we obtained in the absence of lipids. In the presence of lipids, the center of gravity of two adjacent GTPase domains was 14.4 nm, which is in accordance with the height of 10 nm we observed for the GTP-bound DRP1 oligomers.

DRP1 is the main mediator of mitochondrial fission. Similar to earlier studies, DRP1 like dynamin is composed of a mechanochemical core of GTPase, a middle, and GTPase effector domain regions. In place of the pleckstrin homology domain in dynamin however, DRP1 contains an unstructured variable domain whose function is not yet fully resolved [54]. Studies have shown that variable domain limits premature DRP1 assembly in solution and promotes membrane curvature. Its absence from the mechanochemical core of DRP1 is sufficient to mediate GTP-hydrolysis dependent membrane constriction [53]. During mitochondrial division, DRP1 is recruited from the cytosol to the outer mitochondrial membrane by one or several integral membrane proteins. One such DRP1 partner protein Mff, is essential for mitochondrial division, but its mechanism of action remains unexplored. Several recent studies have emphasized the role of Mff interaction with DRP1, where dimeric DRP1 is selectively recruited by Mff to initiate assembly of a functional fission complex [53, 54, 69].

One striking difference between the molecular arrangement of human DRP1 and dynamin 1 is the arrangement of the domains with respect to the center of the oligomers, presumably reflecting a ~90° rotation of the monomer towards the membrane. For Yeast Dnm1, the GTPase domain is located on the outside, the PH domain closer to the membrane and the GED/MID domain and stalk, in the middle [20, 22, 42]. For DRP1, the GTPase and GED/MID domains are located at the same distance from the center of the oligomer or the membrane (S1 and S3 Figs). It should also be noted that even in the presence of lipids, no densities contacting the membrane were detected in the EM map of Yeast Dnm1 that could be attributed to the B-insert or VD domain [9]. While this may be due to domain flexibility, a similar arrangement between Yeast Dnm1 and DRP1 cannot be excluded.

DRP1 and Yeast Dnm1 lack the predicted PH lipid-binding domain of dynamin 1. In its place, DRP1 and Yeast Dnm1 harbor an uncharacterized B-insert or VD domain. Similar to the previous study of Yeast Dnm1, we were unable to resolve the position of the B-insert in our 3D structure [9]. There is only 24% amino acid similarity between the Yeast Dnm1 and DRP1 variable domains. Moreover, this domain has been found to be superfluous for mitochondrial recruitment, association with the DRP1 anchoring protein Mff, or mitochondrial fragmentation [29]. This domain has, however, an autoinhibitary role for mitochondria fission, a role regulated by phosphorylation [29]. Some factors have been shown to activate Yeast Dnm1/DRP1 GTPase activity and self-assembly. They may function as conformational nucleators by inducing or stabilizing the GTP conformation. One of these factors in yeast is Mdv1 that interacts with Yeast Dnm1 and Fis1 [70].

Upregulation of DRP1 plays a critical role in mitochondria on a string (MOAS) and tubulation phenotype, which are often found in pathological conditions such as stroke, brain trauma and altered brain metabolism associated with progression of Alzheimer’s disease (AD) [71–73]. Similar to these findings, we have shown in our in vitro studies that DRP1 is directly involved in the tubulation of mitochondria. We have recently shown that mutant Huntingtin proteins play a similar role with DRP1 by decreasing the lag phase of assembly and GTP hydrolysis [4]. On the basis of this data, it is highly unlikely that the B-insert mediates interaction between adjacent monomers. It is, however, possible that its conformation that is induced by phosphorylation and/or binding of activation factors may affect the position of the other domains, such as the BSE, and may influence self-assembly.

In the current model of membrane constriction by dynamin, Yeast Dnm1, and DRP1, GTP hydrolysis mediates constriction of the oligomers and brings the membranes that are meant to be fused closer together [9, 42, 54]. It should be noted, that none of the previously observed constricted conformations allow two membranes to be close enough to undergo spontaneous fission and fusion. The oligomers that we observed for DRP1 had inner diameters of 20 nm; the diameter of a mitochondria is 0.5 − 1.0 μm. This means that substantially greater remodelling is needed for mitochondrial fission than for the membrane fission of vesicles. An alternative model is, therefore, oligomerization upon DRP1 binding of GTP, which would be sufficient to constrict mitochondria and induce membrane fusion (Fig 7) (S3 Fig).

In the presence of GTP or GNP-PNP in vitro, DRP1 tubulates mitochondria by forming rings or short helices around the mitochondria, to mediate fission (Fig 6). Such observations suggest the existence of an intricate signaling mechanism whereby mitochondria tubulate in the presence of endogenous adapter proteins like Mff, MiD49, and MiD5 and helping DRP1 to bind to its receptor, leading to constriction and fission [48, 53, 54, 69]. Sites of DRP1 short helices binding on the elongated tubular area suggest the theory of spiral helices of DRP1 tubulating and constricting mitochondria [53, 54]. In in vivo conditions, endoplasmic reticulum (ER) and actin seem to be required for signalling early stage of DRP1 recruitment on the mitochondrial membrane, but in in vitro condition, this step is compensated with excess DRP1[74–76]. Mitochondrial tubulation does not initially need dynamin 2, but is required for the final fission steps [77, 78].

Our model proposes multiple sites on the mitochondria where DRP1 binds, forming helices to tubulate the organelle. The final constriction for fission may, however, require a GTP-dependent oligomerization of a ring-like assembly (Fig 7). DRP1 mediates mitochondrial membrane constriction to a tubular form. The regulated recruitment of dynamin 2, in presence of other factors, completes the membrane-tubule constriction to the point of fission[77]. The complex of DRP1 fission factors, adaptor proteins, and nucleotide constricts synergistically the mitochondria and forms a ring at its least stable position.

## Supporting information

S1 Fig Domain comparison of human dynamin with human DRP1 isoform 3. (DOCX)

S2 Fig Top view of 6 classes (13–18 monomer) of DRP1 oligomeric rings (Scale bar = 30 nm). (DOCX)

S3 Fig A) **Current model of dynamin self-assembly.** Dynamin dimers are formed through the interactions of their stalk domains (shown in blue). Two dimers assemble by the interaction of the GTPase domains (shown in green). The PH domain (shown in pink) binds and inserts into the lipid membrane (Chappie *et al*., 2011). **B**) **Our proposed model for DRP1 self-assembly**. The DRP1 monomers assemble in a parallel fashion with respect to the membrane, stalk and GTPase domains, which are equidistant from the membrane. The interaction of DRP1 with the lipid membrane is shown in plan-view (left cartoon) and side-view (right cartoon).
(DOCX)

S1 Table Sequence comparison of human DRP1 (NM_005690.3) with Yeast Dnm1 (P54861), Human dynamin 1 (Q05193), Rat dynamin 1 (P21575), human MxA (P20591), DRP1 Arabidopsis thaliana (3T34). (DOCX)

S2 Table Comparison of human DRP1 rings and helices bound to nanogold in the absence and presence of Immidazole. (DOCX)
