# Supplementary material for: Molecular mechanism of DRP1 assembly studied in vitro by cryo-electron microscopy
Source: PLoS One. 2017 Jun 20;12(6):e0179397. doi: 10.1371/journal.pone.0179397 (PMC5478127; doi:10.1371/journal.pone.0179397)
Supplement: S1 Fig — (DOCX) [file pone.0179397.s001.docx]

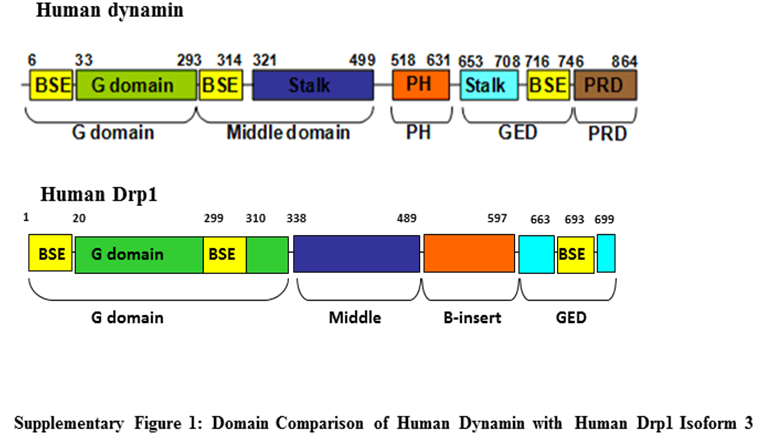


6

33

293

314

321

499

518

631

653

708

716

746

864

1

20

299

310

338

489

597

663

693

699


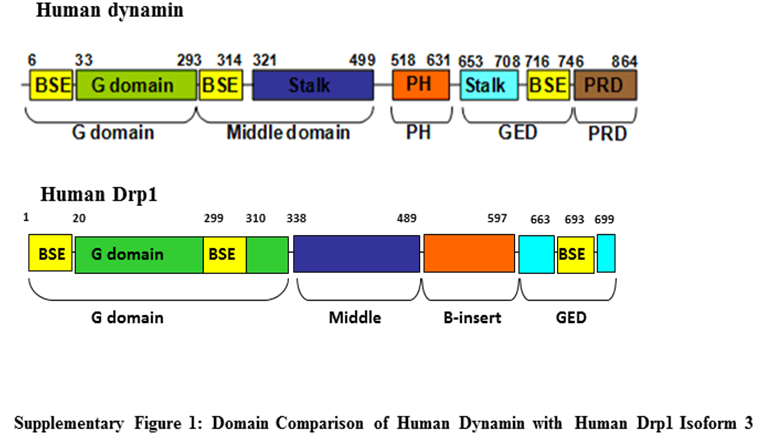


**Human Dynamin**

**Human Drp1**

**G domain**

**Middle domain**

**PH**

**GED**

**PRD**

**G domain**

**Middle domain**

**B-insert**

**GED**

**S1 Fig.**
