# Supplementary figures and images for: Molecular mechanism of DRP1 assembly studied in vitro by cryo-electron microscopy
Source: PLoS One. 2017 Jun 20;12(6):e0179397. doi: 10.1371/journal.pone.0179397 (PMC5478127; doi:10.1371/journal.pone.0179397)

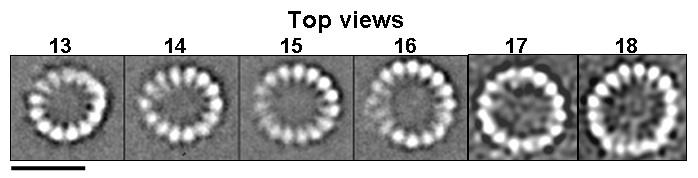


**S2 Fig.**

Supplement: S2 Fig — (DOCX) [file pone.0179397.s002.docx]

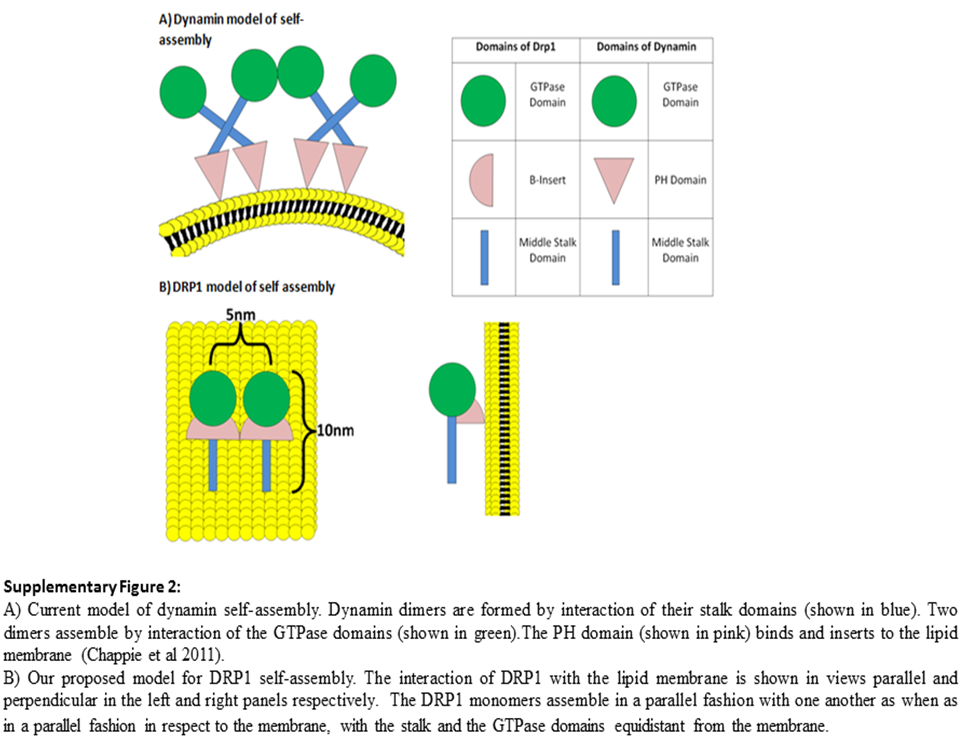


**S3 Fig.**

Supplement: S3 Fig — A) Current model of dynamin self-assembly. Dynamin dimers are formed through the interactions of their stalk domains (shown in blue). Two dimers assemble by the interaction of the GTPase domains (shown in green). The PH domain (shown in pink) binds and inserts into the lipid membrane (Chappie et al., 2011). B) Our proposed model for DRP1 self-assembly. The DRP1 monomers assemble in a parallel fashion with respect to the membrane, stalk and GTPase domains, which are equidistant from the membrane. The interaction of DRP1 with the lipid membrane is shown in plan-view (left cartoon) and side-view (right cartoon). (DOCX) [file pone.0179397.s003.docx]

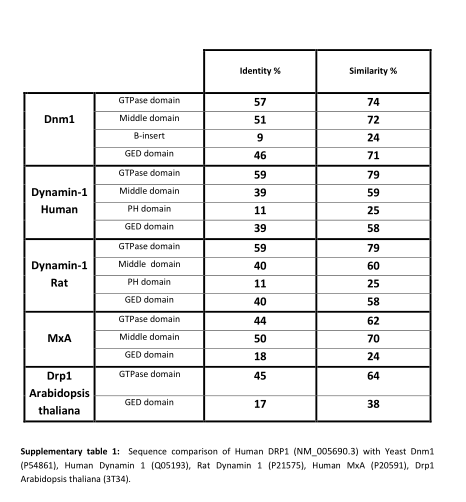


**S1 Table**

Supplement: S1 Table — (DOCX) [file pone.0179397.s004.docx]
