# Supplementary material for: Molecular mechanism of DRP1 assembly studied in vitro by cryo-electron microscopy
Source: PLoS One. 2017 Jun 20;12(6):e0179397. doi: 10.1371/journal.pone.0179397 (PMC5478127; doi:10.1371/journal.pone.0179397)
Supplement: S2 Table — (DOCX) [file pone.0179397.s005.docx]

|  | **Rings without gold** | **Rings with inside gold** | **Rings with outside gold** |
| --- | --- | --- | --- |
| **No. of DRP1 Rings** | **111 (74.00%)** | **28 (18.66%)** | **11 (7.33%)** |
| **No. of DRP1 Rings with 40 mM Immidazole** | **23 (52.77%)** | **18 (40.92%)** | **3 (6.81%)** |
| **No. of DRP1 Helices** | **139 (34.32%)** | **244 (60.24%)** | **22 (5.40%)** |
| **No. of DRP1 Helices with 40 mM Immidazole** | **310 (64.44%)** | **169 (35.13%)** | **2 (0.41%)** |

**S2 Table**
